# Oral Immunization With a M Cell-Targeting Recombinant *L. Lactis* Vaccine LL-plSAM-FVpE Stimulate Protective Immunity Against *H. Pylori* in Mice

**DOI:** 10.3389/fimmu.2022.918160

**Published:** 2022-07-07

**Authors:** Le Guo, Furui Zhang, Shue Wang, Runle Li, Lele Zhang, Zhen Zhang, Runting Yin, Hongpeng Liu, Kunmei Liu

**Affiliations:** ^1^ Department of Medical Laboratory, School of Clinical Medicine, Ningxia Medical University, Yinchuan, China; ^2^ Ningxia Key Laboratory of Clinical and Pathogenic Microbiology, General Hospital of Ningxia Medical University, Yinchuan, China; ^3^ Key Laboratory of Radiation Oncology of Taizhou, Taizhou Hospital of Zhejiang Province affifiliated to Wenzhou Medical University, Taizhou, China; ^4^ Research Center for High Altitude Medicine, Qinghai University, Xining, China; ^5^ Cancer Hospital, General Hospital of Ningxia Medical University, Yinchuan, China; ^6^ School of Pharmacy, Jiangsu University, Zhenjiang, China; ^7^ Ningxia Key Laboratory of Cerebrocranial Diseases, Ningxia Medical University, Yinchuan, China

**Keywords:** *H. pylori*, virulence factor, recombinant *L. lactis* vaccine, M cell-targeting, surface display system

## Abstract

There are many virulence factors of *H. pylori* that contribute in diverse ways to gastric disease. Therefore, designing multivalent epitope vaccines against many key virulence factors virulence factors of *H. pylori* is a promising strategy to control *H. pylori* infection. In previous studies, we constructed a multivalent epitope vaccine FVpE against four key virulence factors of *H. pylori* (Urease, CagA, VacA, and NAP), and oral immunization with the FVpE vaccine plus a polysaccharide adjuvant (PA) containing *lycium barbarum* polysaccharide and chitosan could provide protection against *H. pylori* infection in the Mongolian gerbil model. Oral vaccines have many advantages over injected vaccines, such as improved safety and compliance, and easier manufacturing and administration. However, the harsh gastrointestinal (GI) environment, such as gastric acid and proteolytic enzymes, limits the development of oral vaccines to some extent. Oral vaccines need a gastrointestinal delivery system with high safety, low price and promoting vaccine antigen to stimulate immune response in the gastrointestinal mucosa. Lactic acid bacteria are gastrointestinal probiotics that have unique advantages as a delivery system for oral vaccines. In this study, a M cell-targeting surface display system for *L. lactis* named plSAM was designed to help vaccine antigens to stimulate effective immune responses in the gastrointestinal tract, and a M cell-targeting recombinant *L. lactis* vaccine LL-plSAM-FVpE was constructed by using the surface display system plSAM. recombinant *L. lactis* vaccine LL-plSAM-FVpE could secretively express the SAM-FVpE protein and display it on the bacterial surface. Moreover, experimental results confirmed that LL-plSAM-FVpE had an enhanced M cell-targeting property. In addition, LL-plSAM-FVpE had excellent M cell-targeting property to promote the phagocytosis and transport of the antigen SAM-FVpE by gastrointestinal M cells. More importantly, oral immunization of LL-plSAM-FVpE or SAM-FVpE plus PA can stimulate IgG and sIgA antibodies and CD4^+^ T cell immune responses against four virulence factors of *H. pylori* (Urease, CagA, VacA, and NAP), thus providing protective immunity against *H. pylori* infection in mice. The M cell-targeting recombinant *L. lactis* vaccine against various key *H. pylori* virulence factors could be a promising vaccine candidate for controlling *H. pylori* infection.

## Introduction

*Helicobacter pylori* (*H. pylori*) infects approximately half of the global populatHion, resulting in various gastric diseases including chronic gastritis, peptic ulcer and gastric cancer (1). Among developing and developed Countries, the prevalence rate of *H. pylori* infection varied from as low as 18.9% in Switzerland to 87.7% in Nigeria (2). Current clinical therapies for *H. pylori* infection are mainly based on a variety of antibiotics, such as clarithromycin, metronidazole and levofloxacin (3). However, antibiotic therapies are facing enormous challenge of *H. pylori* resistance to multiple antibiotics (4). Vaccination has been considered as the most promising strategy to control *H. pylori* infection. However, so far there is no commercial *H. pylori* vaccine available.

To date, many *H. pylori* virulence factors have been identified and characterized. Neutrophil-activating protein (NAP) is a virulence factor of *H. pylori*, which activates neutrophils and promote gastric inflammation (5). *H. pylori* NAP has been demonstrated to be an effective vaccine immunogen in both prophylactic and therapeutic vaccine against *H. pylori* infection (6, 7). Urease is critical for *H. pylori* colonization and survival in the stomach, and *H. pylori* tolerance to gastric acid is highly dependent on urease (8). Urease has been widely used in *H. pylori* vaccine design and diagnosis (9–11). CagA is the main virulence factor of *H. pylori*, which can be delivered into gastric epithelial cells by the type IV secretion system (T4SS) of *H. pylori* (12). Moreover, CagA is identified as the first identified bacterial oncoprotein which plays a critical role in *H. pylori*-induced gastric carcinogenesis (13). *H. pylori* can produce and secrete a major toxin, VacA, which contributes to *H. pylori* colonization and virulence in a variety of ways (14). For example, VacA can disrupt mitochondrial functions and inhibit the activation and proliferation of T lymphocytes (15). VacA has been identified as a promising vaccine antigen, especially detoxified VacA (16). Indeed, many virulence factors are involved in the pathogenesis of *H. pylori.* Therefore, a multivalent vaccine against a variety of key virulence factors of *H. pylori* is more likely to provide effective protection than a univalent vaccine targeting only one *H. pylori* virulence factor.

Oral vaccine has been considered to be an attractive vaccine against many gastrointestinal pathogens, such as enterohemorrhagic *E. coli*, *Vibrio cholerae* and *H. pylori*. Oral vaccine is preferable to traditional injection-based vaccine for several reasons, such as improved safety and easier manufacturing. Moreover, oral vaccination can stimulate humoral and cellular immune responses at mucosal sites (17). However, oral vaccination is challenging, requiring appropriate adjuvants or delivery systems to overcome the harsh environment and barriers in the gastrointestinal (GI) tract (18). The currently licensed human oral vaccines primarily use attenuated viruses or pathogenic bacteria as delivery carriers. While these vaccines can effectively stimulate a strong mucosal immune response, the attenuated viruses or pathogenic bacteria as delivery carriers have the risk of reversion to virulence (19). Moreover, the attenuated viruses or pathogenic bacteria cannot be used as delivery carriers in immunologically sensitive populations. Development of Lactic acid bacteria (LAB) as a delivery carrier of oral vaccine is very attractive. LAB have several unique advantages as a delivery carrier of oral vaccine including: gastric acid resistance, stability, activation of both innate and adaptive immunity, and the generally recognized as safe (GRAS) status (20). So far, LAB as a delivery carrier of oral vaccine has been explored against many viral and bacterial pathogens (21, 22). Because of probiotic properties of LAB and the requirements for mucosal vaccines, strategies for the display of vaccine antigens at the surface of LAB are gaining increasing interest.

A prerequisite for successful oral vaccines is that oral vaccine antigens should be devoured and transported into the mucosa-associated lymphoid tissue (MALT) across the mucosal barrier by Microfold cells (M cells). Consequently, development of adjuvants or delivery systems for oral vaccines on the basis of an understanding of antigen uptake and transport mechanism of M cells has attracted substantial research interest. In fact, M cell targeting has been attempted by using various M cell-targeting ligands, such as M cell-specific antibodies, Co1 (23), Cpe17 (24) and CKS9 (25). However, many challenges still remain, such as the identification and design of new M cell-specific ligands, and the discovery of an effective mucosal adjuvant or a new mucosal immune delivery system.

In our previous studies, we constructed a multivalent epitope vaccine FVpE against four virulence factors of *H. pylori* (Urease, CagA, VacA, and NAP), and oral immunization with FVpE plus polysaccharide adjuvant (PA) could provide protective immunity against *H. pylori* infection (26). Here, a M cell-targeting *L. lactis* surface display system plSAM was designed and used for assisting the FVpE vaccine to induce effective immune responses in the GI tract, and a M cell-targeting recombinant *L. lactis* vaccine LL-plSAM-FVpE was successfully constructed. The immunological efficacy of LL-plSAM-FVpE in the prevention and treatment of *H. pylori* infection was evaluated in mice.

## Materials and Methods

### Construction of M Cell-Targeting *L. Lactis* Surface Display System

The M cell-targeting *L. lactis* surface display system plSAM is a designed *L. lactis* plasmid. The plSAM plasmid contains a core component named SAM, which consists of the signal peptide (SPusp45), the propeptide (PS), the multiple clone site (MCS), a designed M cell-targeting peptide (Mtp), and the peptidoglycan-binding domain of AcmA (cA domain). The SAM gene was synthesized, and then inserted into the plasmid pNZ8148 to produce the plasmid plSAM.

### Construction of Recombinant *L. Lactis* Vaccine LL-plSAM-FVpE

The FVpE vaccine is composed of NAP, three selected fragments (CagA_302−437_, VacA_1−46_ and VacA3_32−494_), and Urease multi-epitope peptide (UE) from CTB-UE (6, 10), as shown in **Supplementary Figure 2A**. To obtain the plasmid plSAM-FVpE, the gene sequence of *H. pylori* multivalent epitope vaccine FVpE was amplified by PCR, and then inserted into the plSAM plasmid. Finally, the plasmid plSAM-FVpE was transformed into *L. lactis* NZ9000 to obtain the recombinant *L. lactis* vaccine LL-plSAM-FVpE.

### Expression of Recombinant *L. Lactis* Vaccine LL-plSAM-FVpE

LL-plSAM-FVpE was cultivated and induced for expression of the SAM-FVpE protein by adding 1 ng/mL nisin. The cellular lysate samples were prepared by centrifugation and sonication. The samples were identified by SDS-PAGE and Western blot using mouse anti-FVpE polyclonal antibody and HRP-labeled Goat Anti-Mouse IgG (Proteintech, USA). In addition, immunofluorescence staining was also used for identify whether LL-plSAM-FVpE can produce the SAM-FVpE protein by using mouse anti-FVpE polyclonal antibody and FITC-labeled Goat Anti-Mouse IgG (Proteintech, USA). **Expression and Purification of the Vaccine Antigen SAM-FVpE in *E. Coli*** The gene sequence of the vaccine antigen SAM-FVpE was inserted into *E. coli* plasmid pCzn1 to obtain the recombinant plasmid peSAM-FVpE. Then, the recombinant plasmid peSAM-FVpE was transformed into *E. coli* ArcticExpress (DE3), and the vaccine antigen SAM-FVpE was purified by Ni2+-NTA affinity chromatography (Bio-Rad, Hercules, CA, USA). **Analysis of Antisera From Mice Orally Immunized With LL-plSAM-FVpE**Specific pathogen free (SPF) BALB/c mice (male, 5-6 weeks old) were assigned at random to 2 groups (n = 6): LL-plSAM-FVpE and LL-plSAM. For LL-plSAM-FVpE group, BALB/c mice were treated by gavage with 300μL of LL-plSAM-FVpE (1 × 10^10^ CFU/mL) on day 1, 2, 8, 9, 15, 16, 22 and 23. For LL-plSAM group, BALB/c mice were orally administered with 300μL of LL-plSAM (1 × 10^10^ CFU/mL) at the same point of time. One week after the last vaccination, blood samples were collected. For ELISA analysis, 96-well microplates were coated with 0.5μg/well of SAM-FVpE, urease, CagA, VacA, NAP or BSA. The antisera from mice immunized with LL-plSAM-FVpE or LL-plSAM were diluted to 1:500.

### Whole-Cell ELISA

Whole-cell ELISA was performed as described previously (27). For testing surface display of the SAM-FVpE protein, the bacterial cells of LL-plSAM-FVpE or LL-plSAM were centrifuged and resuspended in PBS. ELISA plates were coated with different amounts of bacterial cells and the SAM-FVpE protein (0.5 μg/well). After washing three times and blocking with 0.5% BSA, mouse anti-FVpE antiserum (1:1000) was added to the ELISA plates. After washing with PBS three times, the ELISA plates were incubated with HRP-labeled Goat Anti-Mouse IgG. Finally, 100 μl/well of tetramethylbenzidine (TMB) was added, and the reaction was stopped by addition of 50 μl/well of 2 M H2SO4. The absorbance of each well was measured at 450 nm.

### M Cell-Targeting Property Analysis

The M cell-targeting properties of LL-plSAM-FVpE and the SAM-FVpE protein were performed as described previously with slight modifications (28, 29). Briefly, 100 μl of LL-plSAM-FVpE, the SAM-FVpE protein (100μg/ml) or the FVpE protein (100μg/ml) were added into the ileal loops respectively. After incubation, the loops were washed, fixed, and then freeze-sectioned. The sections were stained with rabbit anti-FVpE antibody and Alexa Fluor 647 Goat anti-rabbit IgG antibody (Abcam, UK). M cells were detected by using Alexa Fluor 488 anti-Gp2 monoclonal antibody (MBL, Japan). In addition, Nuclei were also stained with DAPI (Sigma, USA).

### Prophylactic and Therapeutic Immunization

**Figure 1:**
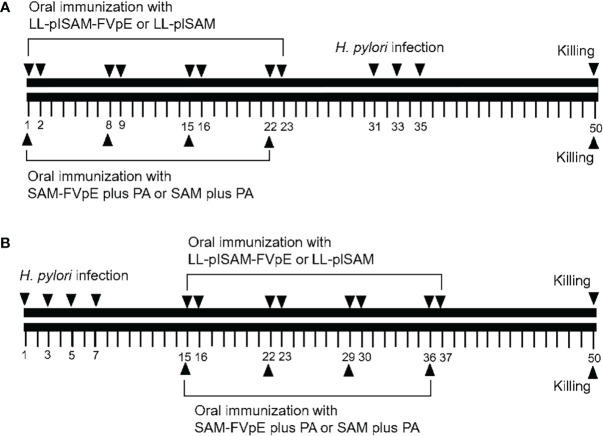
Schematic representation of prophylactic and therapeutic immunization. **(A)** Prophylactic immunization. Mice were administered orally with LL-plSAM-FVpE and LL-plSAM. Moreover, mice were also orally vaccinated with SAM-FVpE or SAM in 500 μl PA adjuvant. After oral vaccination, mice were challenged with (*H*) *pylori* on designated days. **(B)** Therapeutic immunization. (*H*) *pylori*-infected mice were divided into 4 groups (n=10). Mice were orally vaccinated with LL-plSAM-FVpE or LL-plSAM (3 × 10^9^ CFU) respectively. Mice were also orally immunized with 100 μg of SAM-FVpE or SAM in 500 μl PA adjuvant. On day 50, the mice were killed.

For prophylactic immunization (**Figure 1A**), the SPF BALB/c mice (6 to 8 weeks old, male) were randomly divided into 4 groups (n = 10): LL-plSAM-FVpE, LL-plSAM, SAM-FVpE plus PA adjuvant, and SAM plus PA adjuvant. LL-plSAM-FVpE or LL-plSAM was cultivated and induced by adding 1 ng/mL nisin for 6 h prior to oral immunization. For LL-plSAM-FVpE group and LL-plSAM group, BALB/c mice were immunized orally with LL-plSAM-FVpE or LL-plSAM (3 × 10^9^ CFU) respectively on days 1, 2, 8, 9, 15, 16, 22 and 23. In addition, mice were also administered with 100 μg of the SAM-FVpE or SAM protein in 500 μl PA adjuvant containing *lycium barbarum* polysaccharide (20μg/mL) and chitosan (1%, w/w). Mice were administered with 300μL of *H. pylori* suspension on days 31, 33 and 35, and mice were sacrificed on day 50. For therapeutic immunization (**Figure 1B**), *H. pylori*-infected mice were prepared by gavaging with *H. pylori* SS1. After *H. pylori*-infected mice were successfully prepared, the mice were divided into 4 groups (n=10): LL-plSAM-FVpE, LL-plSAM, SAM-FVpE plus PA adjuvant, and SAM plus PA adjuvant. The first 2 groups of mice were orally vaccinated with *L. lactis* strain, LL-plSAM-FVpE or LL-plSAM (3 × 10^9^ CFU) on designated days respectively. The latter 2 groups of mice were orally immunized with 100 μg of the vaccine antigen SAM-FVpE or SAM in 500 μl PA adjuvant on designated days. On day 50, the mice were killed.

### Analysis of *H. Pylori* Infection

After oral vaccination and *H. pylori* challenge, *H. pylori* infection was evaluated by quantitative culture of the bacteria, quantitative PCR (qPCR) and rapid urease test. For quantitative culture of *H. pylori*, samples of gastric tissue were weighed and homogenized. After serial dilution, the tissue homogenate was plated to Columbia agar plates with antibiotics (Qingdao Hope Bio-Technology Co., Ltd.). All colony counts were reported as colony-forming units (CFU) per gram of tissue. For qPCR analysis, *H. pylori* SSA gene was measured, normalized for mouse stomach GAPDH expression, as previously described (30). For rapid urease test (RUT), A piece of gastric tissue sample was immersed in the RUT solution, and incubated at 37°C for 4 hours. The **absorbance** value **was read at 550** nm.

### Gastric Histological Examination

Gastric Histological examination was performed by hematoxylin and eosin (HE) staining, inflammation scores and immunohistochemical (IHC) analysis. Briefly, A piece of gastric tissue was fixed by using 10% neutral formaldehyde solution, and then embedded in paraffin. Sections were stained with HE, and the gastritis was evaluated as previously reported (31). The tissue sections were also observed for the presence of *H. pylori* infection by IHC staining using a rabbit anti-*H. pylori* polyclonal antibody (Linc-Bio, Shanghai, China) (32).

### Analysis of *H. Pylori*-Specific Antibodies

Serum IgG and mucosal secretory IgA (sIgA) were quantified by ELISA. Briefly, microplates were coated overnight at 4°C with 0.5 μg/well of *H. pylori* lysates. Serum IgG and mucosal secretory IgA (sIgA) were detected with HRP-conjugated goat anti-mouse IgG and HRP-conjugated goat anti-mouse IgA (Jackson ImmunoResearch, USA), respectively.

### Analysis of *H. Pylori*-Specific Lymphocyte Responses

Mice were sacrificed, and the spleens were harvested. The spleen lymphocytes were extracted, and cultured with *H. pylori* lysates (5 μg/ml) for 72 h. Cell proliferation was measured by the CCK-8 assay (Dojindo Molecular Technologies, Inc. Japan). To detect the level of cytokines expression, the supernatant of lymphocytes culture was collected after stimulation with *H. pylori* lysates. Cytokines (IL-4, IFN-γ and IL-17) were quantified by using ELISA kits, following the kit instructions.

### Statistical Analysis

Statistical analysis was carried out using GraphPad Prism 5.0 software, and results are presented as mean ± standard deviation (SD). Student’s t test was used to test statistical significance. p <0.05 was considered to be statistically significant.

## Results

### Construction of M Cell-Targeting *L. Lactis* Surface Display System

To obtain M cell-targeting *L. lactis* surface display system, *L. lactis* plasmid plSAM, the core component SAM was synthesized, and inserted into the plasmid pNZ8148 (**Supplementary Figure 1A**). After digestion by Nco I and Hind III, the plasmid plSAM could produce an 887 bp fragment, which roughly equaled to the size of the SAM gene (**Supplementary Figure 1B**). These results confirmed that the plasmid plSAM were successfully constructed.

### Construction of Recombinant *L. lactis* Vaccine LL-plSAM-FVpE

The structure of the multivalent epitope vaccine FVpE is shown in **Supplementary Figure 2A**. The FVpE gene was amplified, and inserted into the plasmid plSAM to obtain the plasmid plSAM-FVpE (**Supplementary Figure 2B**). The plasmid plSAM-FVpE was confirmed by restriction enzyme digestion and gene sequencing (**Supplementary Figure 2C**). Finally, the plasmid plSAM-FVpE was electrotransfered into *L. lactis* NZ9000, and recombinant *L. lactis* vaccine LL-plSAM-FVpE was successfully prepared.

### Expression Analysis of Recombinant *L. Lactis* Vaccine LL-plSAM-FVpE

**Figure 2:**
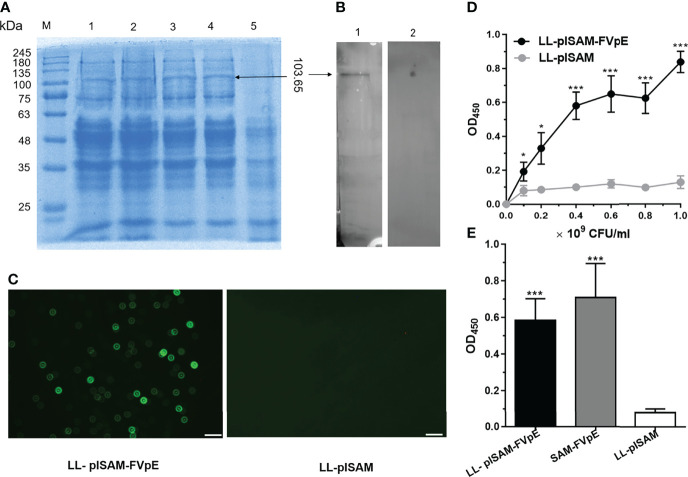
Expression and identification of recombinant *L. lactis* vaccine LL-plSAM-FVpE. **(A)** SDS-PAGE. Protein marker (M); After induction with nisin, the lysate supernatants of LL-plSAM-FVpE (lane 1, 2, 3 and 4). The lysate supernatants of LL-plSAM-FVpE without induction with nisin (lane 5). **(B)** Western blot. The SAM-FVpE protein can be detected by mouse anti-FVpE antibody (lane 1). The SAM-FVpE protein was not detected by normal mouse serum (lane 2). **(C)** Immunofluorescence. LL-plSAM-FVpE or LL-plSAM were stained with mouse anti-FVpE antibody and FITC labeled goat anti-mouse IgG. **(D)** Whole cell ELISA. Different amounts of *L. lactis* LL-plSAM-FVpE or LL-plSAM were immobilized on ELISA plates. The protein SAM-FVpE was detected with mouse anti-FVpE and HRP-labeled Goat Anti-Mouse IgG. *p <0.05, ***p <0.001. **(E)** Whole cell ELISA. The plates were coated with LL-plSAM-FVpE (5 × 10^8^ CFUs/well), LL-plSAM (5 × 10^8^ CFUs/well) and the protein SAM-FVpE (0.5 μg/well). The protein SAM-FVpE was detected with mouse anti-FVpE antiserum and HRP-labeled Goat Anti-Mouse IgG. ***p <0.001.

After induction by nisin, LL-plSAM-FVpE could produce the fusion protein SAM-FVpE (103.65 kDa), as shown in **Figure 2A**. The results from Western blot confirmed that the fusion protein SAM-FVpE could be detected by mouse anti-FVpE polyclonal antibody, but not by normal mouse serum (**Figure 2B**). In addition, LL-plSAM-FVpE could emit green light by immunofluorescence analysis, however, no green light was detected in LL-plSAM (**Figure 2C**). These results suggested that LL-plSAM-FVpE could express the fusion protein SAM-FVpE successfully. In addition, the results from whole-cell ELISA confirmed that the SAM-FVpE protein was displayed on the bacterial surface (**Figures 2D, E**).

### Specificity of Antiserum Specific for LL-plSAM-FVpE and Immunoreactivity of the Vaccine Antigen SAM-FVpE

**Figure 3:**
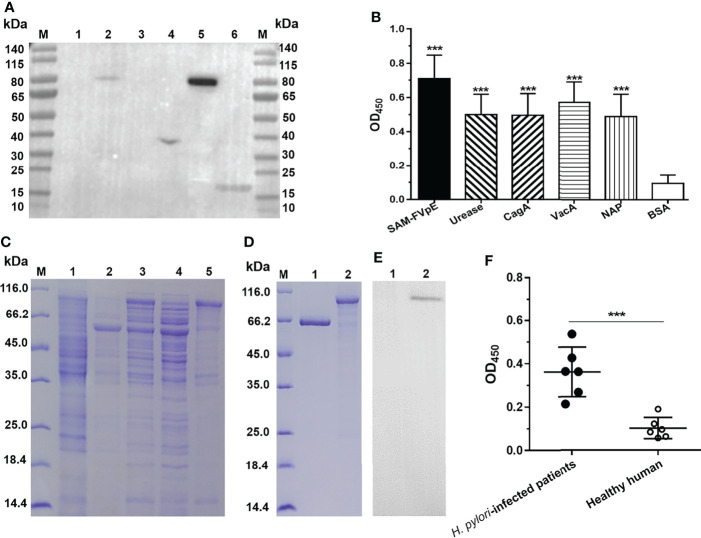
Specificity of antiserum specific for LL-plSAM-FVpE and immunoreactivity of the vaccine antigen SAM-FVpE. Antisera were prepared by oral vaccination of mice with LL-plSAM-FVpE. **(A)** Western blot. The antisera specific for LL-plSAM-FVpE can recognize (*H*) *pylori* antigens. Protein marker (lane M), BSA (lane 1, 3), (*H*) *pylori* urease (lane 2), recombinant CagA (lane 4), VacA (lane 5), and NAP (lane 6). **(B)** ELISA. The plates were coated with 0.5μg/well of SAM-FVpE, urease, CagA, VacA, NAP and BSA. The antisera specific for LL-plSAM-FVpE was diluted to 1:500. ***p <0.001. **(C)** Expression of the vaccine antigen SAM-FVpE in *(E) coli.* Protein marker (lane M), ArcticExpress (DE3)/pCZN1 (lane 1), ArcticExpress (DE3)/peSAM-FVpE without IPTG induction (lane 2), ArcticExpress (DE3)/peSAM-FVpE with IPTG induction (lane 3), the soluble proteins of ArcticExpress (DE3)/peSAM-FVpE with IPTG induction (lane 4), the inclusion bodies of ArcticExpress (DE3)/peSAM-FVpE with IPTG induction (lane 5). **(D)** Purification of the vaccine antigen SAM-FVpE. Protein marker (lane M), BSA (lane 1), SAM-FVpE (lane 2). **(E)** Western blot. The SAM-FVpE proteins could react specifically with antiserum specific for LL-plSAM-FVpE. BSA (lane 1), SAM-FVpE (lane 2). **(F)** ELISA. The sera from (*H*) *pylori*-infected patients could recognize the SAM-FVpE protein. The sera were diluted 100 times. ***p <0.001.

The results from Western blot (**Figure 3A**) and ELISA (**Figure 3B**) confirmed that antiserum specific for LL-plSAM-FVpE could recognize the four *H. pylori* virulence factors (Urease, NAP, VacA and CagA). However, it could not react with the control BSA protein. To obtain large amounts of the vaccine antigen proteins SAM-FVpE with high purity, the SAM-FVpE proteins were also expressed in *E. coli* (**Figure 3C**) and purified by Ni^2+^-NTA affinity chromatography (**Figure 3D**). In addition, the SAM-FVpE proteins could react specifically with antiserum specific for LL-plSAM-FVpE (**Figure 3E**). More importantly, the SAM-FVpE proteins could be recognized by the sera from *H. pylori*-infected patients (**Figure 3F**).

### Detection of M Cell-Targeting Properties

**Figure 4:**
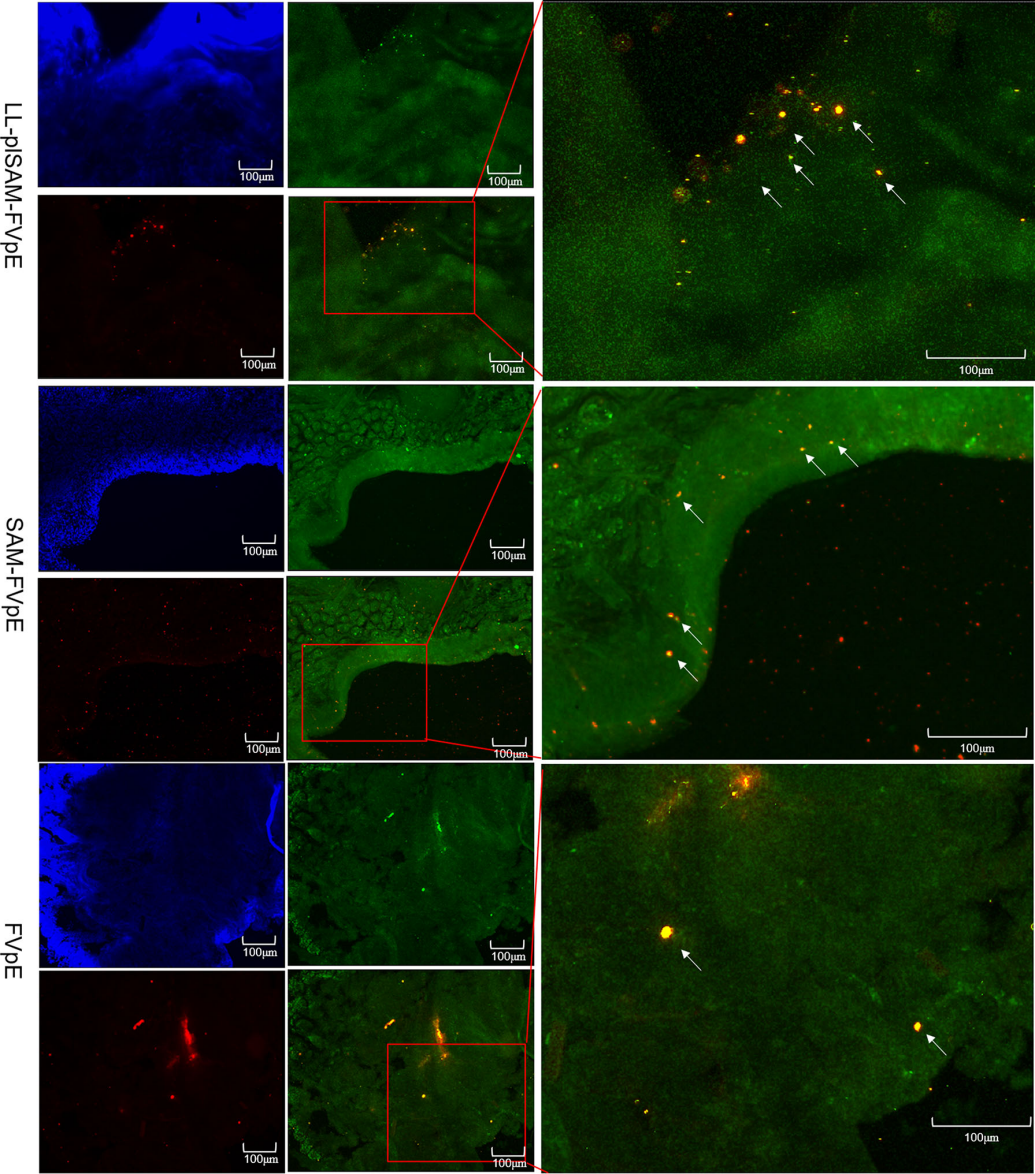
Analysis of M cell-targeting properties. For the ileal loop test, *L. lactis* LL-plSAM-FVpE, SAM-FVpE or FVpE were injected into the loops. White arrows indicate co-localization signals for antigens targeting M cells.

Closed ileal loop assay and immunohistochemistry (IHC) were performed to identify whether LL-plSAM-FVpE and the SAM-FVpE protein have M cell-targeting properties. The *L. lactis* LL-plSAM-FVpE, the SAM-FVpE protein or the FVpE protein were injected into the ileal loops respectively, and the fluorescent signals for FVpE were monitored by using rabbit anti-FVpE antibody and Alexa Fluor 647 Goat anti-rabbit IgG antibody. In addition, a well-known M cell specific antibody, anti-Gp2-FITC, was used to detect M cells in Peyer’s patch. The results revealed that the groups treated with LL-plSAM-FVpE or the SAM-FVpE protein showed much more yellow fluorescent signals in Peyer’s patch compared to the control group treated with the FVpE protein (**Figure 4**), suggesting that *L. lactis* LL-plSAM-FVpE or the SAM-FVpE protein have better M cell-targeting properties owing to the SAM component.

### Prophylactic Effect of Recombinant *L. Lactis* Vaccine LL-plSAM-FVpE

**Figure 5:**
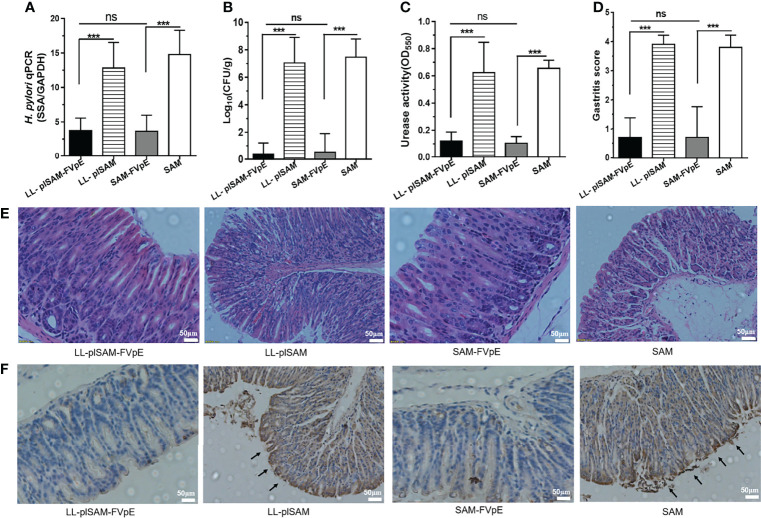
Analysis of protective effects and gastric histopathology. **(A–C)** Mice were administered orally with *L. lactis* LL-plSAM-FVpE, *L. lactis* LL-plSAM, SAM-FVpE plus PA, or SAM plus PA, and then challenged with *H. pylori*. *H. pylori* load in the stomach was analyzed by quantitative culture, qPCR and rapid urease test. ***p <0.001; ns: not significant. **(D, E)** Moreover, gastric histopathological analysis was performed by HE staining and scoring of gastritis. Gastric tissues from mice immunized with LL-plSAM-FVpE or SAM-FVpE plus PA have lower inflammatory scores compared with mice immunized with LL-plSAM or SAM plus PA. Typical pictures of HE staining. Gastric tissue from mice vaccinated with LL-plSAM or SAM plus PA, exhibiting severe inflammatory infiltrates (100×). **(F)** IHC staining. There were clusters of *H. pylori* colonization in gastric tissue samples from mice administered with LL-plSAM or SAM plus PA.

After oral vaccination and *H. pylori* challenge, the bacterial load of *H. pylori* in the stomach was analyzed by quantitative culture, qPCR and rapid urease test. The gastric examination by quantitative culture of *H. pylori* (**Figure 5A**), qPCR (**Figure 5B**) and rapid urease test (**Figure 5C**) showed that oral immunization with LL-plSAM-FVpE or SAM-FVpE plus PA significantly reduced *H. pylori* burden and urease activity compared with LL-plSAM or SAM plus PA. More importantly, eight out of ten mice found no *H. pylori* colonization in the stomach. Gastric histopathological analysis was further performed by HE staining, scoring of gastritis and immunohistochemistry (IHC). The results from HE staining and scoring of gastritis showed that oral vaccination with LL-plSAM-FVpE or SAM-FVpE plus PA could significantly reduce stomach inflammation in mice compared with LL-plSAM or SAM plus PA. Moreover, no significant difference in the extent of stomach inflammation was detected between the LL-plSAM-FVpE group and the SAM-FVpE plus PA group (**Figures 5D, E**). The results of IHC analysis showed that the control groups, the LL-plSAM and SAM plus PA groups, had cluster of *H. pylori* colonization in gastric tissue samples. However, both LL-plSAM-FVpE and SAM-FVpE plus PA groups found only small amounts of *H. pylori* or no bacteria in gastric tissue samples, basically confirming the histopathologic observations (**Figure 5F**).

### *H. Pylori*-Specific Lymphocyte and Antibody Responses After Prophylactic Immunization

**Figure 6:**
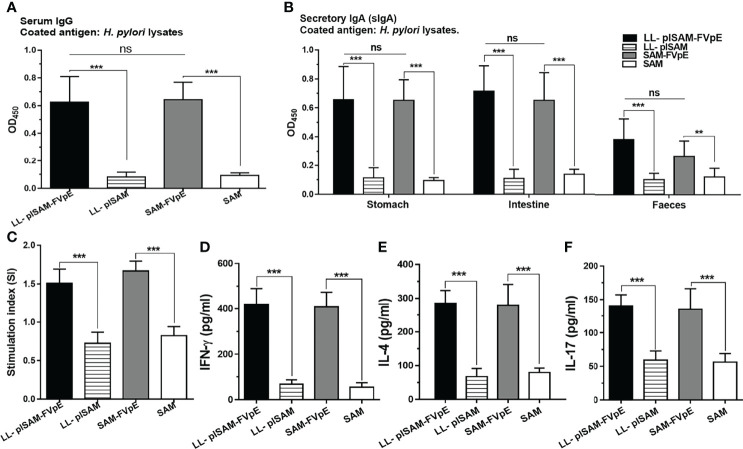
(*H*) *pylori*-specific lymphocyte and antibody responses after prophylactic immunization. **(A, B)** Serum IgG and mucosal sIgA against (*H*) *pylori*. The sera, stomach, intestine and feces were collected. The sera (1:1000 diluted), stomachs and intestine samples (diluted 1:5 in PBS) were tested for (*H*) *pylori*-specific antibodies by ELISA. ***: p <0.001, ns not significant. **(C)** Proliferation of (*H*) *pylori*-specific lymphocytes. Lymphocytes were isolated from mice immunized with LL-plSAM-FVpE, SAM-FVpE plus PA, LL-plSAM or SAM plus PA, and cultured with (*H*) *pylori* lysates (5 μg/ml) for 72 h. Lymphocyte proliferation was determined. ***: p <0.001. **(D–F)** Detection of the cytokines. After stimulation with (*H*) *pylori* lysates, cytokines (IFN-γ, IL-4 and IL-17) were detected by ELISA. ***: p <0.001.

The ELISA plates were coated with 0.5 μg/well of *H. pylori* lysates. The sera, stomach, intestine and fecal samples were tested by ELISA for *H. pylori*-specific antibodies. As shown in **Figures 6A, B**, oral vaccination with *L. lactis* LL-plSAM-FVpE or the SAM-FVpE protein plus PA could stimulate significantly elevated serum IgG and mucosal sIgA antibodies against *H. pylori*. To test the capacity of LL-plSAM-FVpE to induce *H. pylori-*specific lymphocyte responses, splenic lymphocytes were cultured with *H. pylori* lysates. Stimulation of splenic lymphocytes from mice immunized with LL-plSAM-FVpE or SAM-FVpE plus PA displayed significantly high proliferation than stimulation of cells from mice immunized with LL-plSAM or SAM plus PA (**Figure 6C**). Moreover, ELISA assays of cytokines in the supernatant of splenic lymphocyte cultures showed that the group immunized with LL-plSAM-FVpE or SAM-FVpE plus PA had significantly elevated IFN-γ (**Figure 6D**), IL-4 (**Figure 6E**) and IL-17 (**Figure 6F**) levels, compared with the group vaccinated with LL-plSAM or the SAM plus PA.

### Therapeutic Effect of Recombinant *L. Lactis* Vaccine LL-plSAM-FVpE

**Figure 7:**
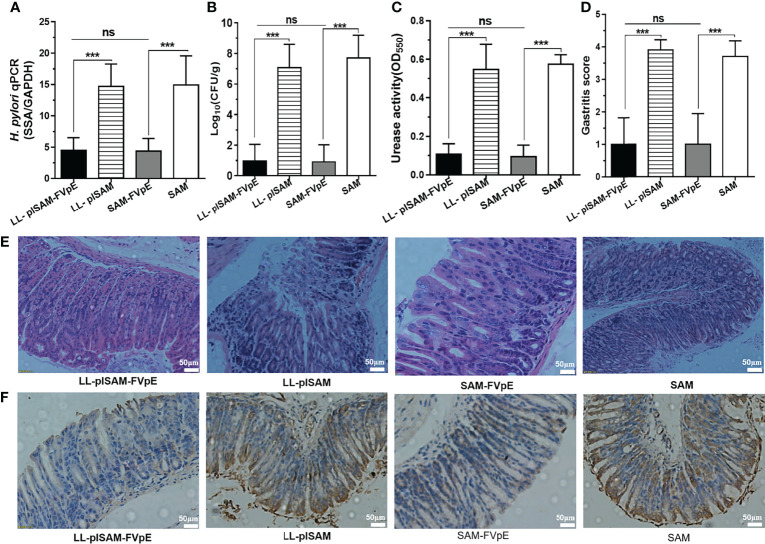
The therapeutic effect of oral immunization with recombinant *L. lactis* vaccine LL-plSAM-FVpE. **(A)** qPCR. Data are expressed as the ratio of (*H*) *pylori* SSA gene to mouse GAPDH. ***p <0.001; ns: not significant. **(B)** Quantitative culture of bacteria. After therapeutic immunization, the amount of CFU in the mouse stomach was determined by quantitative culture of bacteria. ***p <0.001; ns: not significant. **(C)** Urease activity test. After therapeutic immunization, (*H*) *pylori* urease activity in the stomach of mice was examined by a rapid urease assay. ***p <0.001; ns: not significant. **(D, E)** Gastritis grading of gastric tissue and representative histopathology images. After therapeutic immunization, inflammation in the stomach of mice was evaluated by HE staining and gastritis grading. ***p <0.001; ns: not significant. **(F)** IHC analysis. The colonization of *(H) pylori* in the stomach of mice was examined by IHC staining.

*H. pylori*-infected mice were gavaged with recombinant *L. lactis* vaccine LL-plSAM-FVpE to examine the effect of LL-plSAM-FVpE in removing *H. pylori* infection. Oral immunization with LL-plSAM-FVpE or SAM-FVpE plus PA adjuvant could reduce bacterial load and urease activity in the mouse stomach (**Figures 7A–C**). In addition, compared with LL-plSAM or SAM plus PA adjuvant, LL-plSAM-FVpE or SAM-FVpE plus PA adjuvant could significantly reduce gastric inflammation in mice (**Figures 7D, E**). More importantly, the IHC results confirmed that LL-plSAM-FVpE or SAM-FVpE plus PA adjuvant could eradicate *H. pylori* infection in five out of 10 *H. pylori*-infected mice. However, no eradication effect was found in mice immunized with either LL-plSAM or SAM plus PA adjuvant (**Figure 7F**). The IHC results were consistent with those of *H. pylori* quantitative culture.

### *H. Pylori*-Specific Antibodies and Cellular Immune Responses After Therapeutic Immunization

**Figure 8:**
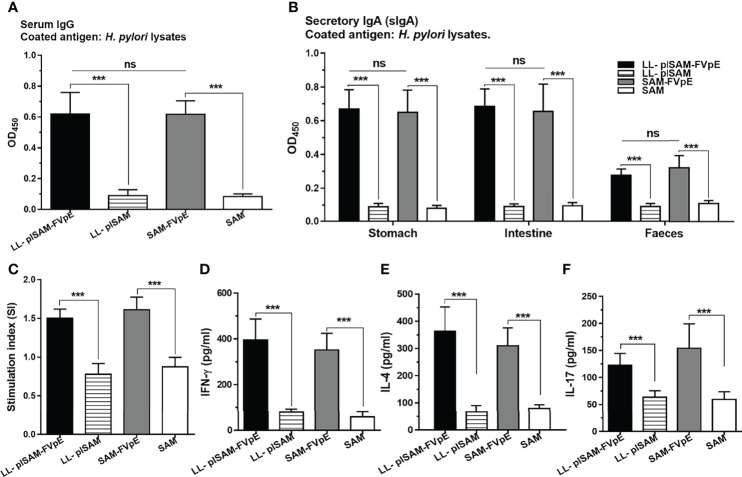
(*H*) *pylori*-specific antibodies and cellular immune responses after therapeutic immunization with LL-plSAM-FVpE. **(A)** Serum IgG antibodies. (*H*) *pylori*-specific serum IgG antibodies were determined by indirect ELISA. ***p <0.001, ns not significant. **(B)** Mucosal sIgA antibodies. (*H*) *pylori*-specific mucosal sIgA antibodies in the gastrointestinal tract were detected by indirect ELISA. ***p <0.001, ns not significant. **(C)** Proliferative response of T lymphocytes. After stimulation with (*H*) *pylori* lysates, the proliferation of splenic T lymphocytes was examined. ***p <0.001. **(D–F)** Analysis of the cytokines. After stimulation with (*H*) *pylori* lysates, cytokines (IFN-γ, IL-4 and IL-17) were analyzed by ELISA. ***p <0.001.

After therapeutic immunization, LL-plSAM-FVpE or SAM-FVpE plus PA adjuvant could stimulate the production of *H. pylori*-specific serum IgG and mucosal sIgA in mice. However, either LL-plSAM or SAM plus PA adjuvant did not excite any *H. pylori*-specific antibodies (**Figures 8A, B**). In addition, after stimulation with *H. pylori* lysates, splenic lymphocytes from mice immunized with LL-plSAM-FVpE or SAM-FVpE plus PA adjuvant could produce significant proliferative responses (**Figure 8C**), and the levels of cytokines (IL4、IFN-γ and IL-17) were significantly increased (**Figures 8D–F**).

## Discussion

*H. pylori* colonizes the gastric mucosa of humans and is associated with many gastric diseases, such as chronic gastritis, peptic ulcer and gastric cancer. *H. pylori* virulence factors, such as urease, the cytotoxin-associated antigen A (CagA), the vacuolating cytotoxin A (VacA) and the neutrophil-activating protein (NAP), play important roles in the development of *H. pylori*-related gastric diseases (33). Urease is essential for colonization and survival of *H. pylori* in the human stomach, which can convert urea into ammonia. *H. pylori* VacA can induce intracellular vacuolation in eukaryotic cells and inhibit T cell responses, which contribute to the persistence of *H* *pylori* infection. CagA is a unique bacterial oncoprotein that is not found in any other bacteria except for *H. pylori*. CagA can be translocated into gastric epithelial cells by *H. pylori* Cag type IV secretion system (Cag-T4SS). Once injected into the gastric epithelial cells, CagA can induce different magnitude of morphological changes, which are associated with the development of gastric cancer.

In our previous studies, a multivalent epitope vaccine FVpE containing NAP, three fragments from CagA and VacA, and urease multi-epitope peptide (UE) from the urease multi-epitope vaccine CTB-UE (10), was constructed. Oral immunization with FVpE could induce *H. pylori* specific CD4^+^ T cell responses and antibodies against urease, CagA and VacA and NAP (32). Despite good immunogenicity and specificity, the FVpE vaccine, as an oral vaccine, still needs a cheap, scale-produced and effective gastrointestinal delivery system. In this study, we designed and constructed a M cell-targeting *L. lactis* surface display system to assist in delivering the vaccine antigen FVpE to gastrointestinal tract, and eventually a M cell-targeting *L. lactis* LL-plSAM-FVpE was obtained. The results confirmed that the SAM-FVpE proteins were expressed on the surface of *L. lactis* LL-plSAM-FVpE, and both LL-plSAM-FVpE and SAM-FVpE had M cell-targeting properties. Mice vaccinated orally with LL-plSAM-FVpE could produce *H. pylori*-specific lymphocyte and antibody responses against urease, CagA and VacA and NAP, and were significantly protected against *H. pylori* infection.

In addition to physical and biological barriers, the gastrointestinal tract is considered as the largest immunological organ. For example, secretory IgA (sIgA) antibodies are predominantly produced at gastrointestinal mucosa and prevent microbial infection by inhibiting gastrointestinal pathogens adhesion to the gastrointestinal tract. The hostile environment of the gastrointestinal tract, such as gastric acid and digestive enzymes, is considered a major obstacle to the development of oral vaccines. To overcome these adverse factors of the gastrointestinal tract, efforts have focused on development of effective oral vaccine delivery systems. Lactic acid bacteria (LAB), such as *L. lactis and Lactobacillus* species, has been developed as oral vaccine delivery systems for oral vaccination (34). Because of the Generally Recognized As Safe (GRAS) status and the long history o use in fermented foods, LAB has been considered to be a safer alternative to live attenuated pathogens such as Salmonella and Mycobacterium. *L. lactis* is a model LAB that has been extensively studied as delivery systems for oral vaccines. So far, *L. lactis* has been used to express various foreign antigens including bacterial (35), viral (36), and parasite antigens (37). Moreover, it has attracted great attention to display vaccine antigens on the surface of *L. lactis* (38). However, most of these studies have simply used *L. lactis* as an expression system, and done relatively little work on the modification of the properties of lactic acid bacteria, such as M-cell targeting property. In this study, a M cell-targeting surface display system for *L. lactis* named plSAM was designed to help vaccine antigens to stimulate effective immune responses in the gastrointestinal tract, and a recombinant *L. lactis* vaccine LL-plSAM-FVpE was constructed. The results of whole-cell ELISA confirmed that the SAM-FVpE proteins were successfully displayed on the surface of LL-plSAM-FVpE. More importantly, oral vaccination with LL-plSAM-FVpE could induce antibodies against multiple *H. pylori* virulence factors (Urease, CagA, VacA and NAP). Moreover, given that M cells are the main gateway to transport luminal antigens to the underlying lymphoid tissues and evoke mucosal immune responses, one promising strategy for oral vaccine development is exploring the potential of M cells by M cell-targeting ligands. The closed ileal loop and IHC assays showed that LL-plSAM-FVpE and the SAM-FVpE protein, both of which contained the M cell-targeting peptide (Mtp), possessed M cell-targeting property. As previously mentioned, M cells are specialized epithelial cells that initiate mucosal immune responses through the uptake and transcytosis of luminal antigens. Therefore, M cell-targeting properties contribute to LL-plSAM-FVpE or SAM-FVpE in stimulating mucosal immune responses. In addition, compared with therapeutic immunization with FVpE plus PA adjuvant in a previous study (26), therapeutic immunization with SAM-FVpE plus PA adjuvant was better at clearing or reducing the amount of *H. pylori* in the stomach of mice, probably due to the presence of SAM component in SAM-FVpE. It should be emphasized that M cell-targeting *L. lactis* surface display system plSAM may be applicable not only for oral vaccines against but also for oral vaccines against other gastrointestinal pathogens. Of course, the immunological efficacy of M cell-targeting *L. lactis* surface display system plSAM to deliver other gastrointestinal pathogens needs further validation.

Development of a vaccine against *H. pylori* infection seems a feasible and promising strategy but no vaccines against *H. pylori* are available to date. Efforts to improve current vaccination strategies for prevention of *H. pylori* infection would greatly benefit from a better understanding of the protective mechanisms. However, the mechanism of protection against *H. pylori* infection has not been fully revealed. Given *H. pylori* colonization in the gastric mucosa, initially it was thought that protection against *H. pylori* infection would be antibody mediated, especially mucosal sIgA. Earlier studies showed that antibody-mediated humoral immunity is important for protection against *H. pylori* infection (39, 40). However, subsequent studies suggested that protection against *H. pylori* infection can occur *via* an antibody-independent mechanism (41, 42). In our study, oral vaccination with LL-plSAM-FVpE could induce specific antibodies against multiple *H. pylori* virulence factors (Urease, CagA, VacA and NAP), which are considered to play important roles in the colonization and pathogenesis of *H. pylori.* More importantly, mucosal sIgA antibodies against *H. pylori* were detected in the gastrointestinal tract after oral vaccination with LL-plSAM-FVpE. Therefore, the protection of LL-plSAM-FVpE against *H. pylori* may be associated with antibody-mediated humoral immunity against multiple virulence factors of *H. pylori*. Most researchers have now found that that CD4+ T cell (Th cell) responses is crucial to the protective immunity against *H. pylori* infection. Protective immunity was obtained by transfer of immune, *H. pylori*-specific Th2 cells, and thus protection was considered to be associated with Th2 cell responses (43). Gastric T cells from *H. pylori*-infected patients exhibit a predominantly Th1 cell responses. However, an increasing number of studies showed that protective immunity against *H. pylori* infection is closely associated with strong Th1 and/or Th17 cell responses (44, 45). NAP is not only a crucial *H. pylori* virulence factor, but also an attractive adjuvant which can promote Th1 responses (46). Thus, NAP was selected as a component of the SAM-FVpE antigen. Moreover, the selected fragments (CagA_302-437_, VacA_1-46_, VacA_332-494_ and UE) in the SAM-FVpE antigen contained many known and predicted CD4^+^ T cell epitopes in order that *L. lactis* LL-plSAM-FVpE expressing the SAM-FVpE antigen could stimulate *H. pylori*-specific CD4^+^ T cell responses against multiple crucial virulence factors of *H. pylori.* We found that splenic lymphocytes from mice immunized with LL-plSAM-FVpE displayed high proliferation after stimulation with *H. pylori* lysates, and the cytokine levels (IFN-γ, IL-17 and IL-4) were increased significantly, indicating that LL-plSAM-FVpE could induce a mixed CD4^+^ T cell response against *H. pylori*. LL-plSAM-FVpE or SAM-FVpE plus PA can be effective in controlling *H. pylori* infection in mice, implying the predicted aggregation fragments of predominant Th or B epitopes (CagA_302−437_, VacA_1−46_ and VacA_332−494_) may also contain mouse Th or B epitopes. Briefly speaking, we consider that the protective immunity of LL-plSAM-FVpE may be associated with specific sIgA and IgG antibodies, and mixed CD4^+^ T cell responses against multiple crucial virulence factors of *H. pylori.* Unfortunately, the immunogenicity and protective efficacy of LL-plSAM-FVpE and SAM-FVpE plus PA are completely identical and comparable. However, recombinant *L. lactis* vaccine LL-plSAM-FVpE can be engineered, mass-produced at a low cost and directly administered orally. Direct oral administration of SAM-FVpE protein is difficult to stimulate potent immune responses. Therefore, we used PA adjuvant containing *lycium barbarum* polysaccharide and chitosan to assist the SAM-FVpE protein in this study. The PA adjuvants are difficult to mass production and of high cost. Although recombinant *L. lactis* vaccine LL-plSAM-FVpE and SAM-FVpE plus PA adjuvant are basically similar in terms of immunogenicity and protective efficacy, recombinant *L. lactis* vaccine LL-plSAM-FVpE is superior to SAM-FVpE plus PA adjuvant in terms of cost, process and mass production.

In summary, a *L. lactis* vaccine LL-plSAM-FVpE against multiple crucial virulence factors of *H. pylori* was constructed, based on a designed M cell-targeting surface display system for *L. lactis. L. lactis* LL-plSAM-FVpE could display the SAM-FVpE antigen on the surface of bacteria, and LL-plSAM-FVpE and the SAM-FVpE antigen had an enhanced M cell-targeting property. Oral immunization with LL-plSAM-FVpE could stimulate antibodies against multiple virulence factors of *H. pylori* (NAP, CagA, VacA and urease) and *H. pylori*-specific CD4^+^ T cells, thus providing protective immunity against *H. pylori* infection. The efficacy of *L. lactis* LL-plSAM-FVpE will be evaluated in other animal models, and clinical trials of a *L. lactis* vaccine against *H. pylori* are expected in the future.

## Data Availability statement

The original contributions presented in the study are included in the article/**Supplementary Material**. Further inquiries can be directed to the corresponding authors.

## Ethics Statement

The studies involving human participants were reviewed and approved by Ethical and Experimental Committee of Ningxia Medical University. The patients/participants provided their written informed consent to participate in this study. The animal study was reviewed and approved by Animal Ethical and Experimental Committee of Ningxia Medical University.

## Author Contributions

Conceived and designed the experiments: LG, KL and HL. Performed the experiments: SW, RL, LZ, FZ, ZZ, RY and HL. Analyzed the data: SW, LG and KL. Contributed reagents/materials/analysis tools: RL and HL. Wrote the manuscript: LG and KL. All authors contributed to the article and approved the submitted version.

## Funding

This work was supported by National Natural Science Foundation of China (32070930, 82160497), Key R & D Plan Project of Ningxia Autonomous Region (2020BFG02012), Natural Science Foundation of Ningxia (2022AAC02034, 2020AAC03154), Science Research Project of Ningxia’s Colleges (NGY2020043), Science and Technology Project of Jiangsu Market Supervision Administration (KJ207561), First-Class Discipline Construction Founded Project of Ningxia Medical University and the School of Clinical Medicine (NXYLXK2017A05), Zhenjiang Social Development Project (SH2020036), Ningxia Innovation And Entrepreneurship Projects for Returnees, Ningxia Youth Top Talent Training Project and “Light of the West” Talent Training Programme of the Chinese Academy of Sciences.

## Conflict of Interest

The authors declare that the research was conducted in the absence of any commercial or financial relationships that could be construed as a potential conflict of interest.

## Publisher’s Note

All claims expressed in this article are solely those of the authors and do not necessarily represent those of their affiliated organizations, or those of the publisher, the editors and the reviewers. Any product that may be evaluated in this article, or claim that may be made by its manufacturer, is not guaranteed or endorsed by the publisher.

## Supplementary Material

Supplementary Figure 1 Construction and identification of the plSAM plasmid. **(A)** Construction of the plasmid plSAM. **(B)** Identification of the plasmid plSAM. 1: the plasmid plSAM. 2: plSAM digested by Nco I and Hind III. M: DNA marker.

Supplementary Figure 2 Construction and identification of the plasmid plSAM-FVpE. **(A)** The structure of the FVpE vaccine. **(B)** Construction of the plasmid plSAM-FVpE. **(C)** Identification of the plasmid plSAM-FVpE. M: DNA marker; 1: the plasmid plSAM-FVpE. 2: Restriction enzyme digestion of the plasmid plSAM-FVpE.
