# Supplementary figures and images for: Oral Immunization With a M Cell-Targeting Recombinant L. Lactis Vaccine LL-plSAM-FVpE Stimulate Protective Immunity Against H. Pylori in Mice
Source: Front Immunol. 2022 Jul 7;13:918160. doi: 10.3389/fimmu.2022.918160 (PMC9336465; doi:10.3389/fimmu.2022.918160)

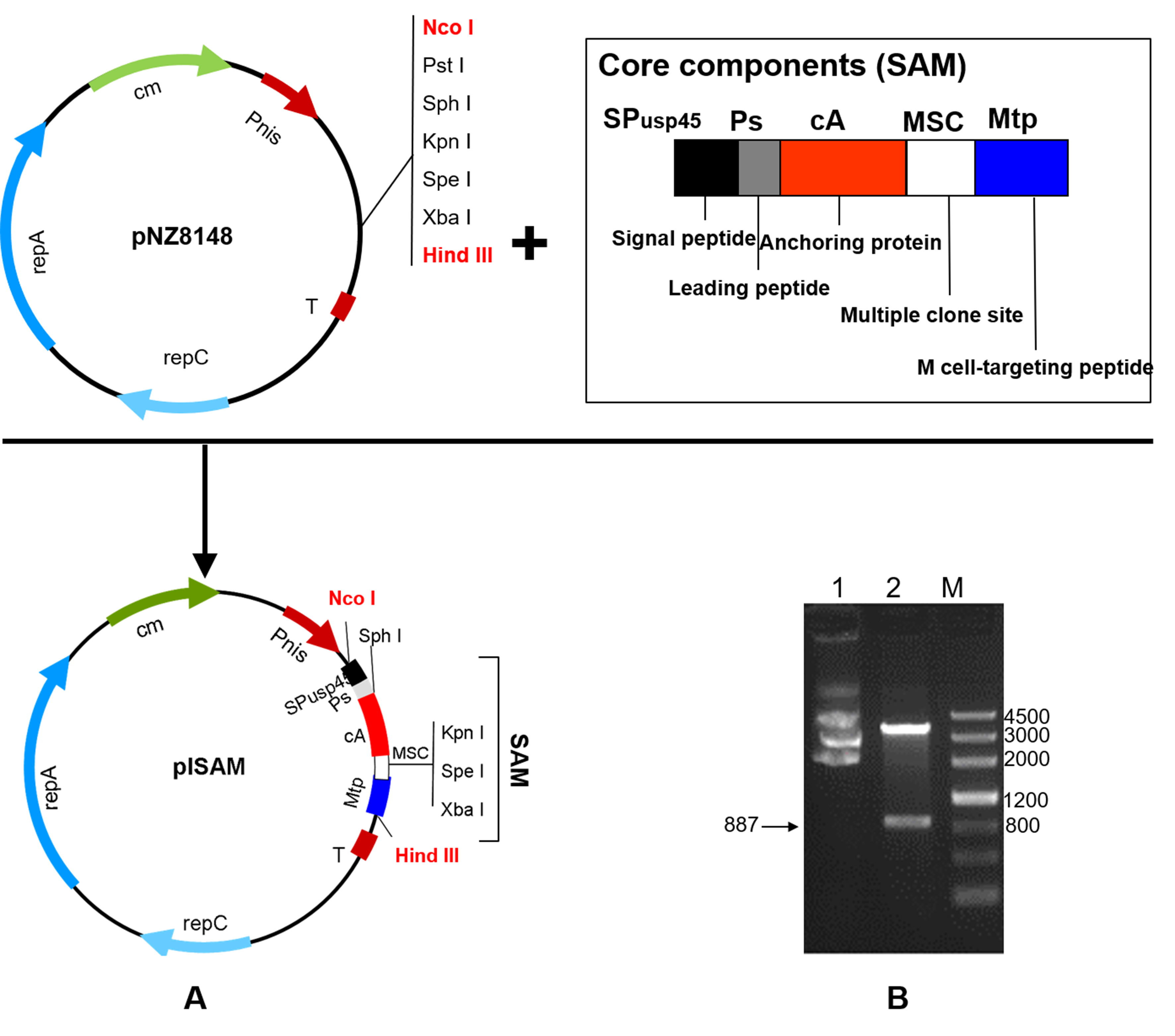

Supplement: Supplementary Figure 1 — Construction and identification of the plSAM plasmid. (A) Construction of the plasmid plSAM. (B) Identification of the plasmid plSAM. 1: the plasmid plSAM. 2: plSAM digested by Nco I and Hind III. M: DNA marker. [file Image_1.tif]

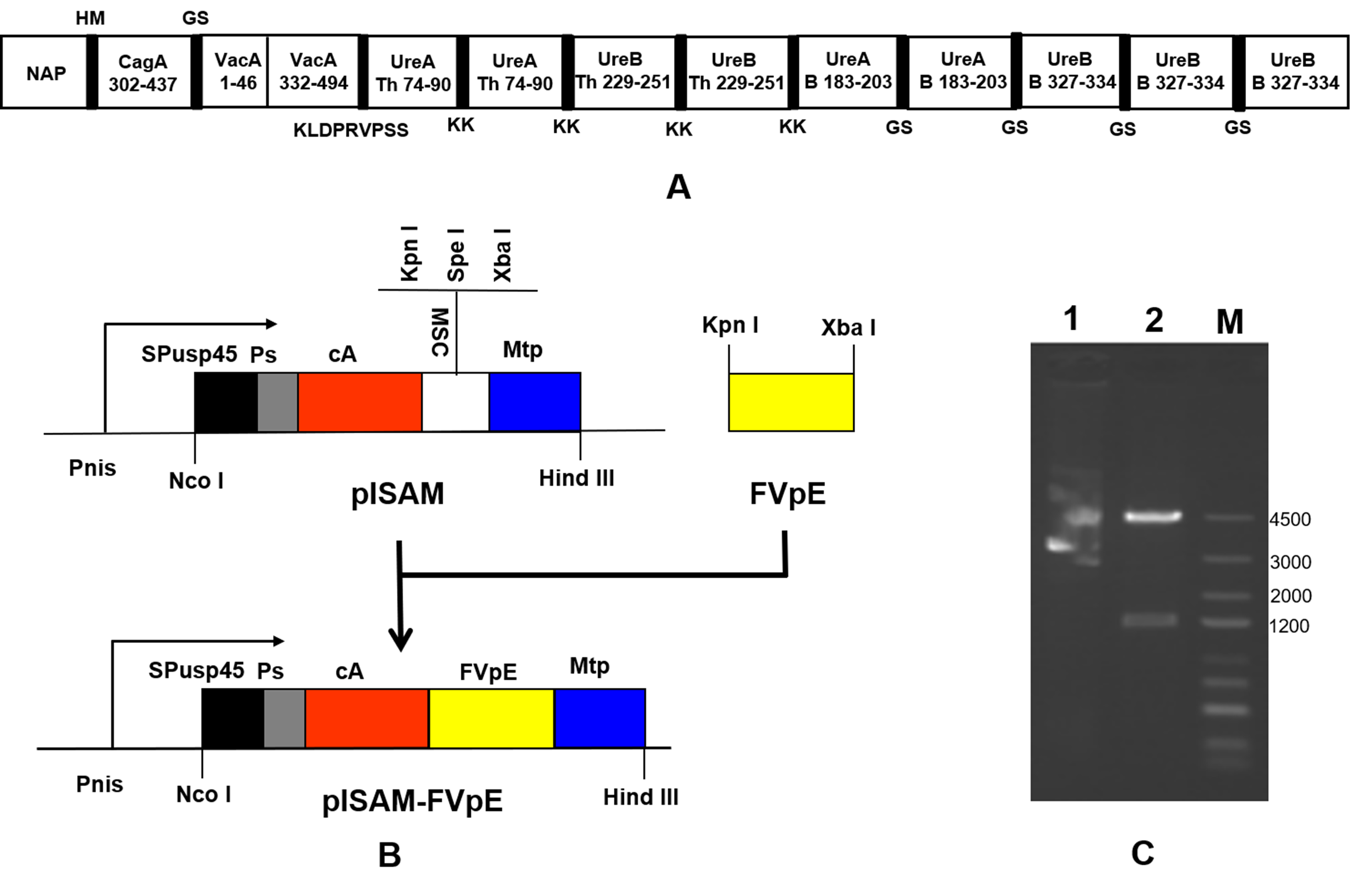

Supplement: Supplementary Figure 2 — Construction and identification of the plasmid plSAM-FVpE. (A) The structure of the FVpE vaccine. (B) Construction of the plasmid plSAM-FVpE. (C) Identification of the plasmid plSAM-FVpE. M: DNA marker; 1: the plasmid plSAM-FVpE. 2: Restriction enzyme digestion of the plasmid plSAM-FVpE. [file Image_2.tif]
